# Temporal profiling of M‐TEER‐related complications

**DOI:** 10.1002/ehf2.15220

**Published:** 2025-01-26

**Authors:** Jafer Haschemi, Hanna Schrameck, Jean Marc Haurand, Daniel Oehler, Maximilian Spieker, Fabian Voss, Malte Kelm, Amin Polzin, Patrick Horn

**Affiliations:** ^1^ Division of Cardiology, Pulmonology and Vascular Medicine, Medical Faculty University of Düsseldorf Düsseldorf Germany; ^2^ Cardiovascular Research Institute, Medical Faculty Heinrich‐Heine University Düsseldorf Germany

**Keywords:** Mitral regurgitation, MitraClip, Pascal, Safety, Events

## Abstract

**Aims:**

Transcatheter edge‐to‐edge repair of the mitral valve (M‐TEER) is known for its low complication rates. However, the optimal level and duration of post‐procedural care remain unclear. This study aimed to identify the specific timeframe of post‐procedural complications following M‐TEER.

**Methods and results:**

We conducted a retrospective analysis of 865 patients who underwent M‐TEER at the University Hospital Düsseldorf between August 2010 and August 2023. Our analysis focused on a comprehensive examination of all acute post‐procedural complications (1–100 h), considering the time point of occurrence or diagnosis. The complication analysed included cardiogenic shock, pericardial tamponade, stroke, cardiac arrhythmias, bleeding, acute kidney injury, myocardial infarction, peripheral vascular ischaemia and in‐hospital mortality.

**Results:**

The median age was 80 (74, 84) years, and the EuroScore II was high (6.5 [4.0, 12.0] %). Functional mitral regurgitation (MR) was more common than degenerative or mixed MR (69% vs. 20%. respectively; 11%). Technical success rate was 97.2%. Overall, acute post‐procedural complications occurred in 87 patients (10.1%). Most complications (75.9%) occurred within the first 4 h post‐procedure. 12.6% of the complications occurred during the period between 4 and 24 h post‐procedure, and 11.5% of the complications happened between 24 and 100 h post‐procedure. Life‐threatening complications were observed only within the first 4 h post‐procedure.

**Conclusions:**

The majority of post‐procedural complications after M‐TEER occur within the first 4 h, with pericardial tamponade and major bleeding occurring only during this period. These findings provide valuable insight for physicians in determining the optimal surveillance and monitoring duration after M‐TEER within clinical settings.

## Introduction

Transcatheter edge‐to‐edge repair of the mitral valve (M‐TEER) has emerged as the standard treatment for patients with clinically relevant mitral regurgitation (MR) and high surgical risk.^1^, ^2^ M‐TEER is a procedure recognized for its safety, with a low complication rate.^3^, ^4^, ^5^ However, while these studies have investigated the overall incidence of complications post‐M‐TEER, the focus has predominantly been on the rates rather than the temporal aspect of their occurrence. Understanding the timing of complications and identifying the associated risk factors is crucial. Such knowledge not only aids in anticipating and promptly addressing complications but also plays an important role in determining the duration and extent of post‐procedural care. This study aimed to assess the temporal occurrence of M‐TEER‐related complications, emphasizing the need to identify the appropriate duration of post‐procedure surveillance. These findings will aid physicians in making well‐informed decisions regarding the appropriate surveillance and monitoring protocols within clinics.

## Methods

We conducted a retrospective analysis of 865 patients who underwent M‐TEER at the University Hospital Düsseldorf between August 2010 and August 2023.

Our analysis focused on a comprehensive examination of all acute post‐procedural complications (occurring between vascular closure of the access site and 100 h post‐procedural). Complications were defined according to the guidelines of the Mitral Valve Academic Research Consortium.^6^ We included conversion to surgery, cardiogenic shock, pericardial tamponade, stroke, myocardial infarction, peripheral ischaemia, cardiac arrhythmias requiring intervention, minor and major bleedings and acute kidney injury (AKI) requiring renal replacement therapy (RRT).

In addition, we assessed late complications (defined as >100 h) and complications not requiring monitoring, such as AKI. We assessed whether these complications occurred during the peri‐procedural or post‐procedural phases of the inpatient stay, considering the time of occurrence or diagnosis. The primary endpoint was time to complication, and the secondary endpoints encompassed the length of hospital stay and 30‐day mortality.

Logistic regression analysis was used to identify variables associated with pericardial tamponade, stroke, cardiac arrhythmias, bleeding, acute kidney injury or in‐hospital mortality after M‐TEER. Variables with a *P*‐value < 0.1 in the univariate analysis, as well as those known or hypothesized to be associated with these complications, were included in the multivariable model when appropriate. Due to the small number of events, cardiogenic shock, myocardial infarction and peripheral ischaemia were not included in the regression analysis.

This study received approval from the local ethics committee of Heinrich Heine University (2019‐558_4) and was conducted according to the principles of the Declaration of Helsinki.

Statistical analyses were conducted using SPSS Statistics (version 28; IBM, Armonk, NY, USA), MedCalc (version 20.11, Ostend, Belgium) and GraphPad Prism (version 9.0; GraphPad Software, San Diego, CA, USA). Categorical variables are presented as absolute values and percentages, whereas continuous data are expressed as medians (interquartile ranges). Categorical data were compared using the χ^2^ test, whereas the D'Agostino and Pearson omnibus normality tests were employed for assessing the normality of the distribution of continuous variables. Student's unpaired *t*‐test (in the case of a normal distribution) or Mann–Whitney *U* test (in the case where continuous variables did not follow a normal distribution) was performed to compare the means between the two groups.

## Results

Between August 2010 and August 2023, 865 patients with MR underwent M‐TEER at the Heart Center Düsseldorf. Baseline characteristics are summarized in *Table* *1*. The median age was 80 (74, 84) years, and the EuroScore II was high (6.5 [4.0, 12.0] %). Functional MR was more common than degenerative or mixed MR (69% vs. 20%, respectively; 11%). Technical success rate of M‐TEER procedure was 97.2% (841 out of 865 patients). The length of hospital stay was 8 (5, 13) days, and the length of intensive care unit (ICU) stay was 1 (1, 2) day (*Table* *2*).

**Table 1:** Patients' characteristics of M‐TEER patients. Values are *n* (%) or median (interquartile range)

Patient characteristics	Complete cohort (n = 865)
Demographics	
Age (years)	80 (74, 84)
Female, *n*/total no. (%)	475/390 (54.9/45.1)
Body mass index (kg/m^2^)	25.4 (22.7, 28.7)
EuroScore II (%)	6.5 (4.0, 15.0)
Elective admission, *n* (%)	735 (85.0)
Comorbidities	
Coronary artery disease, *n* (%)	601 (69.5)
Peripheral arterial disease, *n* (%)	97 (11.2)
Previous cardiac surgery, *n* (%)	302 (34.9)
Previous myocardial infarction, *n* (%)	168 (19.4)
ICD/CRT, *n* (%)	169 (19.5)
Arterial hypertension, *n* (%)	739 (85.4)
Diabetes mellitus, *n* (%)	234 (27.1)
Dialysis for end‐stage renal disease, *n* (%)	19 (2.2)
Chronic lung disease, *n* (%)	158 (18.3)
Atrial fibrillation, *n* (%)	570 (65.9)
Clinical presentation	
NYHA functional class III or IV, *n* (%)	691 (79.9)
MR aetiology, *n* (%)	
Degenerative MR	172 (19.9)
Functional MR	598 (69.1)
Mixed disease	95 (11.0)
Degree of MR at rest, *n* (%)	
3	484 (56)
4	663 (44)
Vena contracta width (mm)	8 (6, 9)
EROA by PISA method (cm^2^)	0.30 (0.24, 0.40)
Regurgitation volume (mL)	47 (40, 64)
LVEF (%)	44 (33, 58)
LVEF >50%, *n* (%)	353 (40.8)
LVEF 40–50%, *n* (%)	191 (22.1)
LVEF <40%, *n* (%)	317 (36.6)
LVEDD (mm)	55 (49, 62)
Left atrial area (mm^2^)	27 (22, 32)
TAPSE (mm)	18 (15, 21)
Systolic PAP (mmHg)	40 (30, 49)
Severe TR, *n* (%)	157 (18)
Cardiac index (l/min/m^2^)	2.0 (1.7, 2.3)
Laboratory parameters	
NT‐proBNP (pg/L)	2384 (1288, 4798)
Haemoglobin (g/dL)	12.3 (10.8, 13.5)
Estimated GFR (mL/min), median	50 (36, 64)

CRT, cardiac resynchronization therapy; EROA, effective regurgitation orifice area; GFR, glomerular filtration rate; ICD, implantable cardioverter defibrillator; LVEDD, left ventricular end‐diastolic diameter; LVEF, left ventricular ejection fraction; MR, mitral regurgitation; NHYA, New York Heart Association; NT‐proBNP, brain natriuretic peptide; PAP, pulmonary artery pressure; RV, regurgitation volume; TAPSE, tricuspid annular plane systolic excursion; TR, tricuspid regurgitation.

**Table 2:** Procedural outcome after M‐TEER. Values are *n* (%) or median (interquartile range)

Procedural outcome	
Technical success, *n* (%)	841 (97.2)
Procedure time (min)	106 (74, 135)
Number of implanted devices	1 (1, 2)
Intraprocedural SLDA, *n* (%)	10 (1.2)
Intraprocedural conversion to surgery	1 (0.1)
Transmitral gradient (mmHg)	3 (3.6, 4.6)
Length of stay (days)	8 (5, 13)
Length of ICU stay (days)	1 (1, 2)
Degree of MR at discharge, *n* (%)	
1+	564 (66.1)
2+	232 (27.2)
3+	46 (5.4)
4+	11 (1.3)

Safety outcome (in‐hospital)	
Peri‐procedural events	
Peri‐procedural mortality, *n* (%)	0 (0)
Cardiogenic shock, *n* (%)	3 (0.4)
Intraprocedural conversion to surgery	1 (0.1)
Myocardial infarction	1 (0.1)
Pericardial effusion, *n* (%)	7 (0.8)
Cardiac arrhythmias, *n* (%)	13 (1.5)
Acute post‐procedural events (0–100 h)	
Pericardial effusion, *n* (%)	3 (0.3)
Cardiac arrhythmias, *n* (%)	14 (1.6)
Stroke, *n* (%)	8 (0.9)
Bleeding, *n* (%)	58 (6.7)
Myocardial infarction, *n* (%)	1 (0.1)
Peripheral ischaemia, *n* (%)	2 (0.2)
AKI requiring RRT, *n* (%)	1 (0.1)
Late events (>100 h) and events not requiring monitoring	
Surgery of mitral valve, *n* (%)	3 (0.3)
AKI not requiring RRT, *n* (%)	81 (9.3)
Death, n (%)	9 (1.0)

AKI, acute kidney injury; ICU, intensive care unit; MR, mitral regurgitation; RRT, renal replacement therapy; SLDA, single‐leaflet device detachment.

### Peri‐procedural complications

Out of 865 patients, 25 (2.9%) experienced peri‐procedural complications (*Table* *2*). Three patients (0.4%) developed cardiogenic shock, all of whom had end‐stage heart failure exacerbated by anaesthesia or mitral valve intervention. One patient (0.1%) required intraprocedural conversion to surgery due to the M‐TEER device being stuck to the left atrial wall. Pericardial tamponade occurred in seven patients (0.8%) and was successfully managed with pericardiocentesis. Cardiac arrhythmias needing intervention (atrial flutter, ventricular tachycardia or heart block) were observed in 13 patients (1.5%). Myocardial infarction, caused by thrombus‐induced occlusion of the right coronary artery, was noted in one patient (0.1%). No peri‐procedural deaths were reported.

### Post‐procedural complications

**Figure 1:**
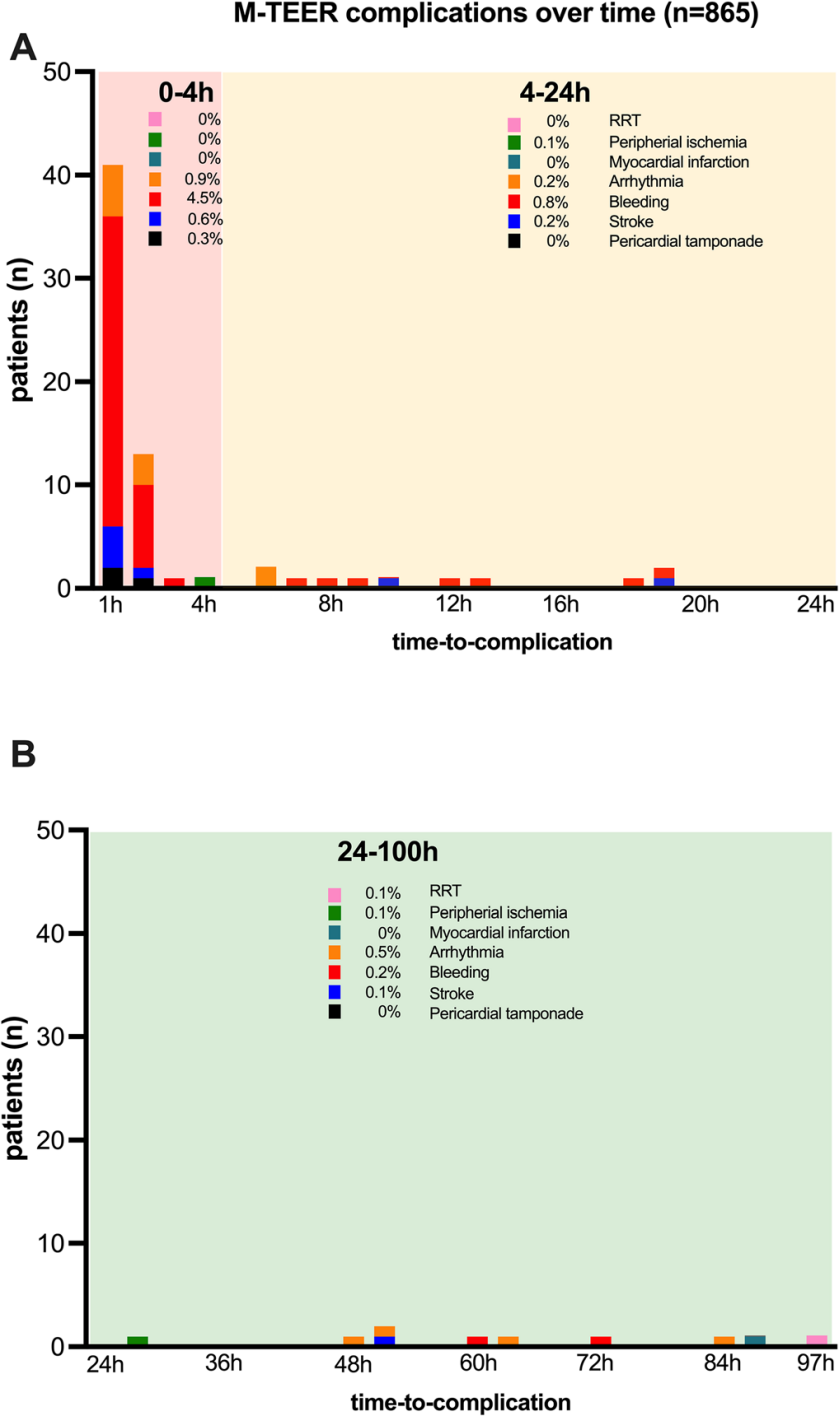
Occurrence of acute M‐TEER‐related complications over time. Occurrence of post‐procedural complication (A) in the first 24 h and (B) between 24 and 100 h after the end of M‐TEER procedure. Post‐M‐TEER time zones are classified according to the timing and severity of complications following the procedure: red zone (0–4 h); yellow zone (4–24 h) and green zone (24–100 h). M‐TEER, transcatheter edge‐to‐edge repair of the mitral valve; RRT, renal replacement therapy.

Acute complications post‐procedure occurred in 87 out of 865 patients (10.1%) (*Table* *2*). Most complications (66 patients, 75.9%) happened within the first 4 h post‐procedure. Complications occurring between 4 and 24 h post‐procedure were seen in nine patients (12.6%), and those occurring between 24 and 96 h post‐procedure were noted in 10 patients (11.5%) (*Figure* *1*).

Pericardial effusion was diagnosed in three patients (0.3%). Two patients developed tamponade, which required drainage, while one patient was treated conservatively.

Stroke occurred in eight patients (0.9%): Five patients (0.6%) were diagnosed within the first 4 h post‐procedure, two patients (0.2%) after 10 and 19 h, respectively, and one patient (0.1%) after 2 days.

Bleeding complications were noted in 58 patients (6.7%): 49 patients (5.7%) experienced bleeding within the first 4 h post‐procedure, predominantly minor access‐site‐related bleeds (90%), seven patients (0.8%) between 4 and 24 h (all minor‐bleedings) and two patients (0.2%) on days 3 and 4 (all minor bleedings).

Cardiac arrhythmias requiring intervention occurred in 14 patients (1.6%). In eight patients (0.9%), arrhythmias were noted during the first 4 h post‐procedure, including one case of atrioventricular block, one case of ventricular tachycardia and six cases of supraventricular tachycardia. Two patients (0.2%) experienced supraventricular arrhythmias between 4 and 24 h, and four patients (0.5%) had supraventricular arrhythmias later (three on day 3 and one on day 4).

Myocardial infarction was reported in one patient (0.1%) on day 4 post‐procedure.

Peripheral ischaemia occurred in two patients (0.2%): one after 4 h and the other on day 2, both induced by a vascular closure device of a femoral arterial sheath.

One patient (0.1%) required initiation of transient RRT after 3 days.

Later during the hospital stay, two patients required mitral valve surgery following M‐TEER failure.

AKI occurred in 82 patients (9.5%): one patient (0.1%) within the first 4 h post‐procedure, nine patients (1.0%) between 4 and 24 h and 72 patients (8.3%) after 24 h.

The in‐hospital mortality rate was 1.0% (9 out of 865 patients), with two deaths due to cardiogenic shock, four due to septic shock, and three due to end‐stage heart failure. Thirty‐day survival status was available for 812 patients, revealing a 2.1% (17 out of 812) mortality rate within the first 30 days.

To identify predictors of specific complications, we performed logistic regression analyses for pericardial tamponade (*Table* *S1*), stroke (*Table* *S2*), bleeding (*Table* *S3*), cardiac arrhythmias (*Table* *S4*), acute kidney injury (*Table* *S5*) and in‐hospital mortality (*Table* *S6*).

In the multivariable analysis, advanced age and reduced left ventricular function were identified as independent predictors of bleeding after M‐TEER (*Table* *S3*). Similarly, worse baseline kidney function and prolonged procedure time were independent predictors of acute kidney injury after M‐TEER (*Table* *S4*).

## Discussion

To the best of our knowledge, this represents the first study to evaluate the time points at which M‐TEER‐related complications occur. We demonstrated that (1) M‐TEER is safe and characterized by a low complication rate; (2) 76% of post‐procedural complications occurred within the first 4 h post‐procedure; and (3) complications as pericardial tamponade and major bleeding occur only within the first 4 h post‐procedure.

In this study, complications during or following M‐TEER were rare, aligning with findings from other comprehensive registries that highlight the comparable safety of M‐TEER.^3^, ^4^, ^5^ When analysing the timeline of complication occurrence and detection, differentiating between peri‐procedural complications promptly detected during vigilant monitoring of the procedure and time‐shifted complications occurring or detected after the procedure is reasonable. Data regarding the timeline of post‐procedural complications will indeed assist physicians in making informed decisions regarding appropriate surveillance and monitoring times in the clinic.

Peri‐procedural complications in M‐TEER can indeed be device‐related, such as single‐leaflet device attachment, partial clip detachment, isolated leaflet damage, clip embolization, conversion to open‐heart surgery and lack of procedural success. Additionally, complications, such as pericardial tamponade, cerebral or coronary air embolism, heart rhythm disorders and cardiogenic shock, may occur.^7^, ^8^ Post‐procedural acute complications include pericardial tamponade, stroke, bleeding, major vascular complications, heart rhythm disorders, acute kidney injury and myocardial infarction.^7^, ^8^

In this study, we emphasized the complications associated with M‐TEER occurring post‐procedure that require direct management or observation. Our particular focus lay in determining the timing and location of these complications. Issues related to the device, such as single‐leaflet device detachment or embolization, are promptly addressed during the procedure but do not constitute the primary focus of our investigation. Our findings demonstrate that the initial 4 h following the conclusion of the M‐TEER procedure present the highest vulnerability. Beyond this timeframe, no pericardial tamponade or major bleeding occurred. Although other types of complications may manifest later, most cases manifest within the initial 4 h. Furthermore, the management of delayed complications, such as AKI not requiring RRT, is not influenced by patient location, whether in the ICU or the general ward.

Based on these data, we propose a conceptual framework in which post‐M‐TEER time zones are classified according to the timing and severity of complications following the procedure:

- Red zone (0–4 h): This signifies the immediate post‐procedural period where the majority of complications occur. Complications within this timeframe are deemed critical (e.g. pericardial tamponade) and necessitate urgent attention and intervention. We recommend surveillance on an ICU or a designated monitoring unit such as a recovery room.
- Yellow zone (4–24 h): This timeframe denotes a period shortly after the procedure but not immediately. Complications within this zone may still require prompt attention, though the urgency might be somewhat less than in the red zone. Prolonged surveillance is advised in the aforementioned higher‐level units or on a regular ward, supported by telemetry for 24 h.
- Green zone (24–100 h): This represents a more delayed post‐procedural period. Complications occurring in this zone are likely to be less urgent (e.g. AKI) but remain important to monitor through laboratory assessments and address. This allows for an extended timeframe for observation and management.

Using these time zones can help healthcare providers prioritize and allocate resources based on the severity and timing of complications. It allows for a more nuanced approach to post‐procedural care, ensuring that immediate attention is given to critical issues while also recognizing the importance of ongoing monitoring for a longer duration.

Moreover, the study data suggest that physicians should consider restricting the duration of ICU stay following M‐TEER. Bypassing or reducing the length of stay in the ICU might be advantageous for patient comfort and well‐being. Furthermore, important treatment objectives, such as early mobilization or maintaining the circadian rhythm, are more attainable outside the ICU. By avoiding the ICU, the total hospital stay can be shortened. This approach not only spares resources in the ICU but also reduces overall costs. However, these potential benefits have to been proven in respective studies.

This study is limited by its single‐centre design and the inclusion of patients over a 13‐year study period. Notably, over the past 6 years, mortality rates have noticeably decreased within the initial 30 days following M‐TEER procedures, indicating overall improvements in outcomes.^5^ This reduction in complications may be attributed to increased experience, meticulous patient selection or changes in vascular closure strategies.

## Conclusions

M‐TEER emerges as a safe procedure characterized by a low complication rate. The majority of post‐procedural complications after M‐TEER occur within the first 4 h, with pericardial tamponade and major bleeding exclusively observed during this timeframe. These findings provide valuable insight for physicians in determining the optimal surveillance and monitoring duration after M‐TEER within clinical settings.

## Conflict of interest

P.H. has received travel support and educational grant from Abbott Medical GmbH and Edwards Lifesciences and an unrestricted research grant from Edwards Lifesciences.

## Funding

Nothing to declare.

## Supporting information


**Table S1.** Regression analysis for pericardial tamponade after M‐TEER.
**Table S2.** Regression analysis for stroke after M‐TEER.
**Table S3.** Regression analysis for bleeding after M‐TEER.
**Table S4.** Regression analysis for cardiac arrhythmias after M‐TEER.
**Table S5.** Regression analysis for acute kidney injury after M‐TEER.
**Table S6.** Regression analysis for in‐hospital mortality after M‐TEER.
