# Supplementary material for: Temporal profiling of M‐TEER‐related complications
Source: ESC Heart Fail. 2025 Jan 26;12(3):2107–12. doi: 10.1002/ehf2.15220 (PMC12055382; doi:10.1002/ehf2.15220)
Supplement: Supplementary file 1 — Table S1. Regression analysis for pericardial tamponade after M‐TEER. Table S2. Regression analysis for stroke after M‐TEER. Table S3. Regression analysis for bleeding after M‐TEER. Table S4. Regression analysis for cardiac arrhythmias after M‐TEER. Table S5. Regression analysis for acute kidney injury after M‐TEER. Table S6. Regression analysis for in‐hospital mortality after M‐TEER. [file EHF2-12-2107-s001.docx]

**Supplemental Table 1.** **Regression analysis for pericardial tamponade after M-TEER**

|  | **Univariate** | | |
| --- | --- | --- | --- |
|  | OR | 95% CI | p-value |
| age | 1.028 | 0.952–1.111 | 0.476 |
| gender | 0.577 | 0.128–2.599 | 0.474 |
| diabetes | 0.614 | 0.129–2.916 | 0.539 |
| CAD | 1.192 | 0.238–5.961 | 0.831 |
| BMI | 0.958 | 0.833–1.103 | 0.553 |
| PAD | 1.228 | 0.146–10.339 | 0.850 |
| CVD | 1.784 | 0.211–15.091 | 0.595 |
| Arial fibrillation | 1.029 | 0.187–5.663 | 0.974 |
| Previous cardiac surgery | 0.395 | 0.047–3.299 | 0.391 |
| EuroSCORE II | 0.918 | 0.815–1.035 | 0.161 |
| Left ventricular ejection fraction | 1.017 | 0.965–1.072 | 0.523 |
| LVEDD | 1.009 | 0.961–1.059 | 0.726 |
| Systolic PAP (mmHg) | 1.009 | 0.966–1.055 | 0.678 |
| TAPSE | 1.116 | 0.944–1.318 | 0.198 |
| GFR | 1.010 | 0.980–1.041 | 0.499 |
| hemoglobin | 1.000 | 0999–1.001 | 0.950 |
|  |  |  |  |
| Brain natriuretic peptide | 0.476 | 0.055–4.086 | 0.499 |
| Procedure time | 1.028 | 0.992–1.066 | 0.131 |

*OR* = odds ratio; *CI* = confidence interval; *CAD* = coronary artery disease; *BMI* = body mass index; *PAD* = peripheral arterial disease; CVD = cerebrovascular disease; *LVEDD* = Left ventricular end-diastolic diameter; *PAP* = pulmonary artery pressure; *TAPSE* = Tricuspid annular plane systolic excursion; *GFR* = glomerular fraction rate

**Supplemental Table 2.** **Regression analysis for stroke after M-TEER**

|  | **Univariate** | | |
| --- | --- | --- | --- |
|  | OR | 95% CI | p-value |
| age | 1.056 | 0.958–1.164 | 0.273 |
| gender | 0.151 | 0.180–1.303 | 0.086 |
| diabetes | 2.486 | 0.497–12.433 | 0.267 |
| CAD | 1.937 | 0.225–16.692 | 0.548 |
| BMI | 0.904 | 0.796–1.027 | 0.122 |
| PAD | 1.001 | 0.123–8.328 | 0.992 |
| CVD | 2.076 | 0.238–18.091 | 0.508 |
| Arial fibrillation | 0.779 | 0.129–4.700 | 0.786 |
| Previous cardiac surgery | 0.474 | 0.055–4.087 | 0.497 |
| EuroSCORE II | 0.964 | 0.893–1.042 | 0.358 |
| Anticoagulation |  |  |  |
| Left ventricular ejection fraction | 1.040 | 0.966–1.121 | 0.299 |
| LVEDD | 0.958 | 0.880–1.043 | 0.321 |
| Systolic PAP (mmHg) | 0.976 | 0.919–1.037 | 0.432 |
| TAPSE | 0.961 | 0.796–1.160 | 0.678 |
| GFR | 0.999 | 0.962–1.038 | 0.957 |
| hemoglobin | 1.000 | 0.999–1.001 | 0.950 |
|  |  |  |  |
| Brain natriuretic peptide | 1.000 | 0.999–1.000 | 0.487 |
| Procedure time | 1.007 | 0.971–1.044 | 0.706 |

*OR* = odds ratio; *CI* = confidence interval; *CAD* = coronary artery disease; *BMI* = body mass index; *PAD* = peripheral arterial disease; CVD = cerebrovascular disease; *LVEDD* = Left ventricular end-diastolic diameter; *PAP* = pulmonary artery pressure; *TAPSE* = Tricuspid annular plane systolic excursion; *GFR* = glomerular fraction rate

**Supplemental Table 3.** **Regression analysis for bleeding after M-TEER**

|  | **Univariate** | | | **Multivariate** | | |
| --- | --- | --- | --- | --- | --- | --- |
|  | OR | 95% CI | p-value | OR | 95% CI | p-value |
| age | 1.037 | 1.001–1.075 | **0.041** | 1.142 | 1.043–1.251 | **0.004** |
| gender | 0.678 | 0.378–1.215 | 0.191 |  |  |  |
| diabetes | 0.646 | 0.319–1.306 | 0.224 |  |  |  |
| CAD | 1.094 | 0.569–2.103 | 0.787 |  |  |  |
| BMI | 0.961 | 0.903–1.023 | 0.209 |  |  |  |
| PAD | 0.931 | 0.375–2.310 | 0.878 |  |  |  |
| CVD | 1.051 | 0.390–2.830 | 0.922 |  |  |  |
| Arial fibrillation | 1.156 | 0.619–2.158 | 0.650 |  |  |  |
| Previous cardiac surgery | 0.828 | 0.437–1.571 | 0.564 |  |  |  |
| EuroSCORE II | 0.990 | 0.967–1.015 | 0.440 |  |  |  |
| Anticoagulation | 1.596 | 0.650–3.924 | 0.308 | 0.904 | 0.279–2.927 | 0.867 |
| Left ventricular ejection fraction | 1.029 | 1.002–1.056 | **0.034** | 1.045 | 1.002–1.090 | **0.039** |
| LVEDD | 0.987 | 0.955–1.021 | 0.453 |  |  |  |
| Systolic PAP (mmHg) | 1.014 | 0.993–1.036 | 0.181 |  |  |  |
| TAPSE | 0.975 | 0.904–1.051 | 0.508 |  |  |  |
| GFR | 0.999 | 0.986–1.012 | 0.870 |  |  |  |
| hemoglobin | 0.853 | 0.731–0.995 | **0.043** | 0.932 | 0.677–1.284 | 0.668 |
|  |  |  |  |  |  |  |
| Brain natriuretic peptide | 1.000 | 1.000–1.000 | 0.669 |  |  |  |
| Procedure time | 1.009 | 0.993–1.025 | 0.282 |  |  |  |

*OR* = odds ratio; *CI* = confidence interval; *CAD* = coronary artery disease; *BMI* = body mass index; *PAD* = peripheral arterial disease; CVD = cerebrovascular disease; *LVEDD* = Left ventricular end-diastolic diameter; *PAP* = pulmonary artery pressure; *TAPSE* = Tricuspid annular plane systolic excursion; *GFR* = glomerular fraction rate

**Supplemental Table 4.** **Regression analysis for cardiac arrhythmias after M-TEER**

|  | **Univariate** | | |
| --- | --- | --- | --- |
|  | OR | 95% CI | p-value |
| age | 0.991 | 0.953–1.032 | 0.673 |
| gender | 2.380 | 0.854–1.624 | 0.097 |
| diabetes | 1.249 | 0.496–3.145 | 0.638 |
| CAD | 1.704 | 0.566–5.133 | 0.344 |
| BMI | 1.001 | 0.975–1.072 | 0.953 |
| PAD | 1.204 | 0.346–4.184 | 0.770 |
| CVD | 1.103 | 0.250–4.864 | 0.897 |
| Arial fibrillation | 0.880 | 0.341–2.269 | 0.792 |
| Previous cardiac surgery | 1.744 | 0.891–2.574 | 0.123 |
| EuroSCORE II | 1.000 | 0.996–1.004 | 0.995 |
| Left ventricular ejection fraction | 0.985 | 0.951–1.020 | 0.404 |
| LVEDD | 1.006 | 0.975–1.038 | 0.710 |
| Systolic PAP (mmHg) | 1.020 | 0.991–1.049 | 0.175 |
| TAPSE | 0.945 | 0.846–1.056 | 0.319 |
| GFR | 0.988 | 0.966–1.011 | 0.293 |
| hemoglobin | 1.000 | 0.999–1.001 | 0.919 |
|  |  |  |  |
| Brain natriuretic peptide | 1.000 | 1.000–1.000 | 0.334 |
| Procedure time | 1.011 | 0.995–1.028 | 0.175 |

*OR* = odds ratio; *CI* = confidence interval; *CAD* = coronary artery disease; *BMI* = body mass index; *PAD* = peripheral arterial disease; CVD = cerebrovascular disease; *LVEDD* = Left ventricular end-diastolic diameter; *PAP* = pulmonary artery pressure; *TAPSE* = Tricuspid annular plane systolic excursion; *GFR* = glomerular fraction rate

**Supplemental Table 5.** **Regression analysis for acute kidney injury after M-TEER**

|  | **Univariate** | | | **Multivariate** | | |
| --- | --- | --- | --- | --- | --- | --- |
|  | OR | 95% CI | p-value | OR | 95% CI | p-value |
| age | 1.010 | 0.961–1.010 | 0.679 |  |  |  |
| gender | 1.339 | 0.520–3.445 | 0.545 |  |  |  |
| diabetes | 1.247 | 0.461–3.374 | 0.663 |  |  |  |
| CAD | 2.145 | 0.618–7.451 | 0.230 |  |  |  |
| BMI | 0.954 | 0.873–1.042 | 0.296 |  |  |  |
| PAD | 0.840 | 0.190–3.708 | 0.818 |  |  |  |
| CVD | 2.151 | 0.603–7.668 | 0.238 |  |  |  |
| Arial fibrillation | 0.655 | 0.241–1.785 | 0.408 |  |  |  |
| Previous cardiac surgery | 1.413 | 0.548–3.648 | 0.474 |  |  |  |
| EuroSCORE II | 1.000 | 0.997–1.003 | 0.891 |  |  |  |
| Left ventricular ejection fraction | 0.989 | 0.951–1.027 | 0.559 |  |  |  |
| LVEDD | 1.006 | 0.970–1.042 | 0.756 |  |  |  |
| Systolic PAP (mmHg) | 1.000 | 0.968–1.034 | 0.977 |  |  |  |
| TAPSE | 0.916 | 0.793–1.058 | 0.233 |  |  |  |
| GFR | 0.873 | 0.834–0.9313 | **0.001** | 0.856 | 0.741–0.989 | **0.034** |
| hemoglobin | 1.000 | 0.991–1.001 | 0.921 |  |  |  |
|  |  |  |  |  |  |  |
| Brain natriuretic peptide | 1.000 | 1.000–1.000 | 0.120 |  |  |  |
| Procedure time | 1.036 | 1.010–1.063 | **0.006** | 1.035 | 1.007–1.063 | **0.014** |

*OR* = odds ratio; *CI* = confidence interval; *CAD* = coronary artery disease; *BMI* = body mass index; *PAD* = peripheral arterial disease; CVD = cerebrovascular disease; *LVEDD* = Left ventricular end-diastolic diameter; *PAP* = pulmonary artery pressure; *TAPSE* = Tricuspid annular plane systolic excursion; *GFR* = glomerular fraction rate

**Supplemental Table 6.** **Regression analysis for in-hospital mortality after M-TEER**

|  | **Univariate** | | | **Multivariate** | | |
| --- | --- | --- | --- | --- | --- | --- |
|  | OR | 95% CI | p-value | OR | 95% CI | p-value |
| age | 1.007 | 0.786–1.007 | 0.786 |  |  |  |
| gender | 0.377 | 0.140–1.147 | 0.154 |  |  |  |
| diabetes | 2.554 | 0.997–6.538 | 0.051 | 0.080 | 0.723–7.123 | 0.999 |
| CAD | 1.869 | 0.531–6.580 | 0.330 |  |  |  |
| BMI | 0.982 | 0.898–1.073 | 0.681 |  |  |  |
| PAD | 1.559 | 0.438–5.551 | 0.493 |  |  |  |
| CVD | 1.673 | 0.982–11.795 | 0.129 |  |  |  |
| Arial fibrillation | 0.926 | 0.307–2.799 | 0.892 |  |  |  |
| Previous cardiac surgery | 2.180 | 0.828–5.740 | 0.114 |  |  |  |
| EuroSCORE II | 1.000 | 0.997–1.003 | 0.955 | 1.052 | 0.910–1.102 | 0.996 |
| Left ventricular ejection fraction | 1.013 | 0.970–1.059 | 0.557 |  |  |  |
| LVEDD | 1.004 | 0.962–1.048 | 0.847 |  |  |  |
| Systolic PAP (mmHg) | 1.021 | 0.984–1.059 | 0.273 |  |  |  |
| TAPSE | 0.996 | 0.867–1.143 | 0.951 |  |  |  |
| GFR | 0.983 | 0.959–1.008 | 0.186 |  |  |  |
| hemoglobin | 1.000 | 0.999–1.001 | 0.992 |  |  |  |
|  |  |  |  |  |  |  |
| Brain natriuretic peptide | 1.001 | 1.010–1.032 | 0.101 |  |  |  |
| Procedure time | 1.034 | 1.010–1.058 | **0.005** | 3.510 | 1.010–1.058 | 0.990 |

*OR* = odds ratio; *CI* = confidence interval; *CAD* = coronary artery disease; *BMI* = body mass index; *PAD* = peripheral arterial disease; CVD = cerebrovascular disease; *LVEDD* = Left ventricular end-diastolic diameter; *PAP* = pulmonary artery pressure; *TAPSE* = Tricuspid annular plane systolic excursion; *GFR* = glomerular fraction rate
